# Characteristics and outcomes of patients with advanced sarcoma enrolled in early phase immunotherapy trials

**DOI:** 10.1186/s40425-017-0301-y

**Published:** 2017-12-19

**Authors:** Roman Groisberg, David S. Hong, Amini Behrang, Kenneth Hess, Filip Janku, Sarina Piha-Paul, Aung Naing, Siqing Fu, Robert Benjamin, Shreyaskumar Patel, Neeta Somaiah, Anthony Conley, Funda Meric-Bernstam, Vivek Subbiah

**Affiliations:** 1 Department of Investigational Cancer Therapeutics (Phase I Clinical Trials Program), Unit 455, Division of Cancer Medicine, The University of Texas MD Anderson Cancer Center, 1515 Holcombe Blvd., Houston, TX 77030 USA; 2 Division of Cancer Medicine, The University of Texas MD Anderson Cancer Center, Houston, TX USA; 3 Department of Sarcoma Medical Oncology, Division of Cancer Medicine, The University of Texas MD Anderson Cancer Center, Houston, TX 77030 USA; 4 Department of Diagnostic Radiology, The University of Texas MD Anderson Cancer Center, Houston, TX 77030 USA; 5 Department of Biostatistics, The University of Texas MD Anderson Cancer Center, Houston, TX 77030 USA

**Keywords:** Sarcoma, Immunotherapy, Checkpoint inhibitor, Phase 1, Anti-PD-1, Anti-PD-L1, Alveolar soft part sarcoma

## Abstract

**Background:**

Immunotherapies, specifically those based on immune checkpoint inhibitors, have shown promising activity in multiple tumor types. Other than mifamurtide (MEPACT®) for osteosarcoma approved by European Medicines Agency, there are no approved immunotherapies for sarcomas.

**Methods:**

We analyzed medical records of patients with advanced sarcoma who were referred to Phase 1 clinic at MD Anderson and received an immunotherapy (checkpoint inhibitors, vaccines, or cytokine based therapies). Clinical parameters including demographics, clinical history, toxicity, and response were abstracted.

**Results:**

Among 50 patients enrolled in immunotherapy trials (Bone 10; Soft-tissue 40) we found 14 different subtypes of sarcomas. Royal Marsden Hospital (RMH) prognostic score was <2 (86%). Performance status (PS) was 0–1 in 48 patients (96%); median number of prior therapies was 3 (0–12). Immunotherapy consisted of checkpoint inhibitors (82%: PD1 = 7, PD-L1 = 11, CTLA4 = 22, other = 1) of which 42% were combinations, as well as vaccines (14%), and cytokines (4%). Median overall survival (OS) was 13.4 months (11.2 months: not reached). Median progression free survival (PFS) was 2.4 months (95% CI = 1.9–3.2 months). Best response was partial response (PR) in 2 patients with alveolar soft part sarcoma (ASPS) and stable disease (SD) in 11 patients (3 GIST, 3 liposarcomas (2 DDLS, 1 WDLS), 2 ASPS, 2 leiomyo, 1 osteo). PFS was 34% (23%, at 50%) at 3 months, 16% (8%, 30%) at 6 months, and 6% (2%, 20%) at 1 year. Pseudo-progression followed by stable disease was observed in 2 patients (4%). Grade 3/4 adverse events included rash (10%), fever (6%), fatigue (6%), and nausea/vomiting (6%).

**Conclusion:**

Immunotherapies were well tolerated in advanced sarcoma patients enrolled in trials. All four ASPS patients had clinical benefit with checkpoint inhibitors and this was the only subtype experiencing partial response. Further evaluation of checkpoint inhibitors in ASPS is warranted.

## Background

Since the approval of anti-CTLA-4 antibody ipilimumab in 2011 and the anti-PD-1 drugs nivolumab and pembrolizumab in 2015, oncology has experienced a resurgence. Patients, clinicians, and drug companies have a new enthusiasm for immunotherapy not seen since the BATTLE trial elevated targeted therapy and small molecules [1]. In the last year melanoma, lung, head and neck, and bladder cancers have shown clinically significant improvement in response rate, progression free survival, and overall survival with the use of the immune checkpoint inhibitors.

Sarcomas are mesenchymal tumors of soft tissues and bone that are usually fatal when they progress beyond local control. Dating back to 1891, attempts have been made to treat sarcomas with immunotherapy [2–5]. These attempts were either with highly toxic “Coley’s toxins” or less potent vaccine therapies as well as unsuccessful trials with interferons. Interestingly, osteosarcoma was one of the first cancers to get regulatory approval with an immunotherapeutic agent. Mifamurtide (L-MTPPE) is an agent that increased circulating TNF-alpha and IL-6. It was approved in Europe for use in combination with adjuvant chemotherapy [6, 7]. Alveolar soft part sarcoma has similarly shown response to immunotherapy with interferon, but only at the case report level [8].

With the advent of modern immune checkpoint inhibitors several trials are ongoing to test the safety and efficacy of immunotherapy in sarcomas. Pre-clinical data suggests that tumor infiltrating lymphocytes (TILs) are an important positive prognostic indicator in multiple soft tissue sarcoma subtypes [9] including angiosarcoma [10] and gastrointestinal stromal tumor (GIST) [11]. Especially with GIST, there is preclinical data to suggest that checkpoint blockade enhances activity of imatinib [12]. PD-L1 expression, which is an important biomarker of response to anti-PD-1 therapy for certain malignancies [13], has been investigated in sarcomas. One study found PD-L1 to be relatively uncommon except in GIST, spindle cell, and radiation associated sarcomas [14]. Others have shown strong expression of PD-L1 in dedifferentiated chondrosarcoma [15], epithelioid, synovial, Ewing, rhabdomyosarcoma [16], and others [17] including bone and leiomyosarcomas [18]. However, as with other sarcoma studies the numbers were small for each subtype. Unfortunately, an early phase II trial of nivolumab in metastatic uterine leiomyosarcomas failed to show a response [19]. However, this may be an issue of patient selection as genomic sequencing has shown lynch syndrome associated gene mutations in a subset of these patients [20]. Other phase II studies have shown partial responses to anti-PD-1 directed therapy in some bone and soft tissue sarcomas [21, 22]. The SARC-028 study in particular showed signals of pembrolizumab activity in undifferentiated pleomorphic sarcoma and dedifferentiated liposarcoma. These two sarcoma cohorts are now undergoing expansion to further evaluate activity [22]. The results have been inconsistent across studies and while the overall trend is that immunotherapy doesn’t have overwhelming activity in sarcomas as observed with melanoma or non-small cell lung cancer, it is clear that patients with certain sarcoma subtypes may respond and investigators continue to evaluate the use of immunotherapy in specific sarcomas such as alveolar soft part sarcoma [23]. We undertook a retrospective review of our own sarcoma patients treated on phase I trials with various immunotherapy agents. The hope is that with more published data on responses, we can begin to intelligently design prospective immunotherapy trials targeted at a specific sarcoma histology or molecular subtype.

## Methods

We reviewed the charts of 50 sequential patients with metastatic or unresectable advanced sarcoma. Pathology had previously been reviewed and verified by an MD Anderson pathologist with expertise in bone and soft tissue sarcomas. All patients had been referred to Investigational Therapeutics Department at MD Anderson Cancer Center (MDACC) and participated in an immunotherapy clinical trial. Trial choice and enrollment was dependent on availability at clinic visit. Immunotherapy was defined as any drug that primes the immune system against the tumor. Our study allowed checkpoint inhibitors, vaccines, and cytokine therapy. Combinations with other drugs were also included. Patient charts were reviewed for age at enrollment on study, race, sex, tumor histology, LDH, albumin, metastatic sites, performance status, prior therapies, toxicity, number of treatment cycles and response based on imaging. Other data collected included the date patient started an investigational therapy, their best response, and duration of that response. Response Evaluation Criteria in Solid Tumors (RECIST V1.1) as well as irRC (immune related response criteria) were used to assess response. Last known follow-up or date of death were also recorded. Progression free survival was measured from date of first dose to date of documented progression on imaging or onset of symptoms. Subsequent scans after progression were reviewed to ensure pseudo-progression was not missed. Toxicities were graded based on NCI CTCAE v4.0.

MD Anderson institutional review board (IRB) independently reviewed and approved each clinical trial presented in our study. Patients were provided with written informed consent prior to treatment with an investigational therapy. MD Anderson IRB also approved this retrospective review.

## Results

**Fig. 1:**
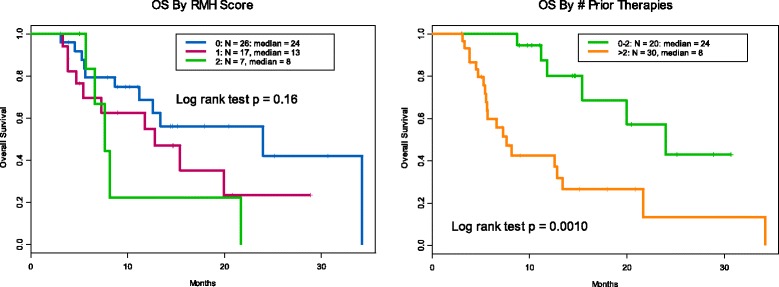
Overall survival by RMH score Hazard Ratio = 2.0 (0.9, 4.6) for 1, 2 vs. 0. “Inconclusive” results. Overall survival by for 0–2 prior therapies median was 24 months and for those with >2 prior therapies was 8 months

Between September 2012 and May 2016 fifty patients with multiple types of sarcoma were enrolled on immunotherapy trials. The clinical characteristics of patients are shown in Table 1. Male to female ratio was 42 to 58%. Median age was 53.5 years (range 18–84). Most patients had favorable RMH score (less than two 43/50, 86%); the remaining 14% of patients had an RMH score of two. Overall survival based on RMH score trended toward favoring lower scores, (Hazard Ratio = 2.0 (0.9, 4.6) for 1, 2 vs. 0, but was statistically inconclusive). Median OS was 24 months for RMH score 0 and was 12 months for patients with score 1–2 (*P* = 0.08). Performance status was also favorable with 48 patients (96%) with an ECOG 0–1. Patients received a median of 3 prior therapies (0–12) and those with two or fewer therapies had improved overall survival compared to those with greater than two prior therapies (*P* = 0.001). (Fig. 1).

**Table 1:** Patient demographics, selected laboratory values, clinical history, and immunotherapy drugs included in this study

Age	
Median	53.5
Range	18–84
Sex	
Female	29
Male	21
No. of metastatic sites	
< 3	37
> =3	13
LDH	
< =ULN	36
> ULN	14
ECOG PS	
0–1	48
2	2
Albumin	
> =3.5 g/dl	46
< 3.5 g/dl	4
RMH score	
< 2	43
> =2	7
Prior treatments	
Median	3
Range	0–12
Anthracycline	30
Ifosfamide	14
Gemcitabine	22
Docetaxel	13
Platinum	8
Investigational therapies	
anti-CCR4 and anti-PD-L1	4
anti-PD-1	7
anti-PD-L1	7
anti-TIM-3	1
anti-CTLA-4	5
anti-CTLA-4 and immunomodulator	7
anti-CTLA-4 and KIT inhibitor	10
Dendritic cell vaccine	7
Interleukin-2	1
anti-TGF-β	1

### Sarcoma subtypes

We found 14 different subtypes of sarcomas with 10 bone sarcomas and 40 soft tissue sarcomas (Table 2).

**Table 2:** Histologic subtypes of sarcomas included in this study

Soft tissue sarcoma	40
Alveolar soft part sarcoma	4
Clear cell sarcoma	2
Desmoplastic small round cell tumor	1
Liposarcoma (Dedifferentiated)	6
Liposarcoma (Well-differentiated)	2
Gastrointestinal stromal tumor (GIST)	9
Leiomyosarcoma	12
Pleomorphic sarcoma	1
Sclerosing Epithelioid fibrosarcoma	1
Solitary fibrous tumor	1
Uterine carcinosarcoma	1
Bone sarcoma	10
Chondrosarcoma (High Grade, III)	4
Ewing sarcoma	1
Osteosarcoma	5

### Therapy

Checkpoint inhibitor based trials made up of single agents in addition to combinations comprised the majority of trials (41/50, 82% (PD1 = 7, PD-L1 = 11, CTLA4 = 22, other = 1). Other therapies included vaccines (7/50, 14%) and cytokines (2/50, 4%). Ten sarcoma patients were enrolled in a clinical trial with ipilimumab and imatinib. (Table 1) Radiation therapy was used in combination with ipilimumab for five patients.

### Adverse events

Adverse events as graded by Common Terminology Criteria for Adverse Events (CTCAE) Version 4.0 included rash (5/50, 10%), fever (3/50, 6%), fatigue (3/50, 6%), and nausea/vomiting (2/50, 4%). Other toxicities included grade 3 hypothyroidism, grade 3 transaminitis, grade 4 pancreatitis, pituitary hypophysitis, pneumonitis and mucositis.

### Responses

**Fig. 2:**
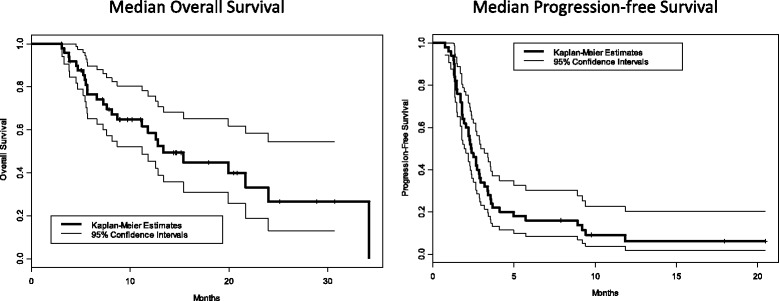
Median OS = 13.4 months, 95% CI = (11.2, not reached); 26 patients died after median 15 months follow-up; OS was 76% (65%, 90%) at 6 months, 59% (45%, 76%) at 1 year, and 27% (13%, 54%) at 2 years (Left). Median PFS = 2.4 months, 95% CI = (1.9, 3.2); 46 patients progressed; PFS was 34% (23%, at 50%) at 3 months, 16% (8%, 30%) at 6 months, and 6% (2%, 20%) at 1 year. (Right)

The median overall survival was 13.4 months, 95% CI = (11.2, not reached). Twenty-six patients died after a median 15 months’ follow-up. Overall survival was 76% (65%, 90%) at 6 months, 59% (45%, 76%) at 1 year, and 27% (13%, 54%) at 2 years. The median progression free survival was 2.4 months, 95% CI = (1.9, 3.2). Forty-six patients progressed; progression free survival was 34% (23%, at 50%) at 3 months, 16% (8%, 30%) at 6 months, and 6% (2%, 20%) at 1 year. (Fig. 2).

**Fig. 3:**
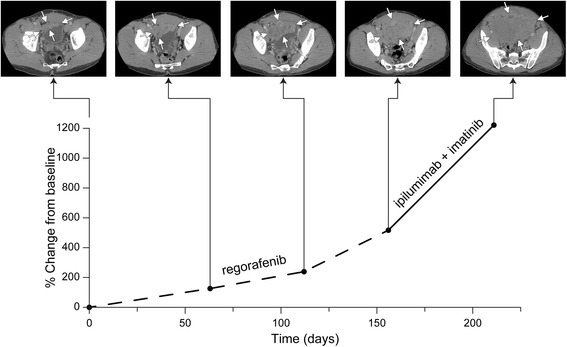
GIST patient with hyper-progression on immunotherapy (based on ir RECIST)

Best response (Table 3) was partial response in 2 patients (4%); both patients had alveolar soft part sarcoma. These two patients both received anti-PD-L1 based therapy (Table 4). Stable disease was observed in 11 patients (22%) with a median of 7.4 months (1.3 to 9.1). GIST (3/ 9 GIST, 33%), well-differentiated liposarcoma (1/2 WDLS, 50%), de-differentiated liposarcoma (2/5 DDLS, 40%), leiomyosarcoma (2/12 LMS, 17%), and osteosarcoma (*n* = 1/5 osteo, 20%) represented patients with stable disease. PFS was 34% (23%, at 50%) at 3 months, 16% (8%, 30%) at 6 months, and 6% (2%, 20%) at 1 year. One of the ASPS patients with stable disease received the same combination as the two partial responders; the other received single agent anti-PD-1. The patient with osteosarcoma received anti-CTLA-4 and radiation; the patients with DDLS received anti-PD-1 or anti-PD-L1; the patients with LMS received anti-CTLA-4 or anti-PD-1. Some patients were treated past progression (16/50, 32%). Pseudo-progression followed by stable disease per ir-RECIST was observed in 2 patients (4%, overall 12.5% of patients treated beyond progression). Both were well-differentiated liposarcomas. One patient with GIST had hyper-progression after treatment with a checkpoint inhibitor. He had continually growing disease prior to therapy, but his rate of tumor growth accelerated upon initiation of immunotherapy (Fig. 3).

**Table 3:** Summary of responders to immunotherapies in this study. Percentage is the number of responders out of the total number treated with that particular histologic subtype

Sarcoma type	Best response	# responders	total	%
Alveolar soft part	Partial response	2	4	50.0%
Alveolar soft part	Stable disease	2	4	50.0%
GIST	Stable disease	3	9	33.3%
Well-diff liposarcoma	Stable disease	1	2	50.0%
De-diff liposarcoma	Stable disease	2	5	40.0%
Leiomyosarcoma	Stable disease	2	12	16.7%
Osteosarcoma	Stable disease	1	5	20.0%

**Table 4:** Brief case history of alveolar soft part sarcomas that responded to immunotherapy

Age	Diagnosis	Prior Therapies	Best response and Duration of response with Immunotherapy
33	Alveolar Soft Part	Sunitinib, Pazopanib, cabozantinbi, Vandetanib/Everolimus,	Partial Response ×12 months
32	Alveolar Soft Part	Cediranib, Sunitinib	Partial Response ×8 months

## Discussion

Metastatic, relapsed and refractory sarcomas continue to have a grave prognosis. There is considerable enthusiasm for developmental therapeutics in sarcomas with recent approvals of pazopanib, eribulin, trabectedin and olaratumab with doxorubicin. There are multiple trials ongoing with the combinations of these agents [24–26]. The exceptional success of immunotherapy in other cancer types spurred us to examine our own records for potential responses to immunotherapy in sarcoma patients. As is frequently the case with sarcomas, our dataset is small and has many different sarcoma subtypes. Admittedly, this mix of low-grade and high-grade sarcomas makes comparison difficult. Complicating matters are the multiple different immunotherapies. Certain observationsemerge even in this heterogeneous group of sarcomas and therapies.

The most remarkable response was that of alveolar soft part sarcomas (ASPS) to immunotherapy. Even with a limited sample of four patients, half had a strong partial response bordering on complete response. The other two patients had stable disease. This is far outside the normal behavior for a biologically indolent but relentless tumor [27] and raises the question of mechanism. Is this a question of PD-L1 blockade and cytotoxic T-cell activation? We know that most tumors with FDA approved anti-PD-1 immunotherapy have response rates in the 10–20% range. This would imply that either our four patients are unusual responders such as those seen in prior interferon trials, or that other mechanisms exist. Tanaka et al. [28] created a mouse model of alveolar soft part sarcoma based on the characteristic *ASPSCR1-TFE3* fusion protein. The model demonstrated a highly vascular tumor with genes expressed in transendothelial migration. This vascularity is key to the early metastatic potential of this tumor. Additionally, ASPS lines these new blood vessels with hemangiopericytes that prevent leakage of nutrients and oxygen out of the blood vessels. We know that chemokines and their ligands are often involved in vascular recognition and targeting of microvascular endothelial cells [29]. Perhaps chemokines play an important role in the action of immunotherapy in ASPS; our group is undertaking further studies to elucidate this mechanism. Alternatively, the TFE3 fusion may be immunogenic itself or act via TGF-β or CD40 ligand to stimulate T-cells and antigen presenting cells [30]. Others have reported that mismatch repair pathway aberrations may be responsible for ASPS response to immunotherapy [31].

Another interesting observationwas seen in the patients with stable disease. It is entirely possible that some of the patients simply had indolent disease, such as the GIST and well-differentiated liposarcoma. However, osteosarcoma, dedifferentiated liposarcoma, and leiomyosarcoma are generally not considered indolent diseases and their stabilization in response to immunotherapy may serve as an indication of activity. While next generation sequencing (NGS) data was not available for the liposarcoma or leiomyosarcoma patients, clinical grade NGS was performed on the osteosarcoma patient. This testing did not reveal a particularly high mutational load which is thought to increase response to immunotherapy. The response of the patients in our study along with recently reported abstracts of positive anti-PD-1 activity in diverse sarcomas suggests that earlier immunotherapy trials in sarcomas were not entirely correct in their negative experience. For example, a recently completed phase II trial of pembrolizumab showed activity in undifferentiated pleomorphic sarcoma and dedifferentiated liposarcoma [32]. Another trial with advanced soft tissue sarcomas treated with pembrolizumab and metronomic cyclophosphamide yielded only one responder out of 50 treated patients [33]. While immunotherapy in sarcomas has shown small promise, we can say that it is unlikely to be the success that it has been in melanoma and non-small cell lung cancer.

GIST is a tumor with great preclinical data for immunotherapy that did not materialize into results for patients. Patients were enrolled on a trial of imatinib and ipilimumab based on convincing pre-clinical rationale that showed imatinib reduced levels of indoleamine 2,3-dioxygenase (Ido). Ido is an immunosuppressive enzyme and inhibition of Ido led to regulatory T-cell destabilization, deactivation, and apoptosis. Treatment naïve mice with KIT mutant GIST treated with imatinib showed decreased regulatory T cell activity [34]. In the clinical trial testing this hypothesis, the combination of imatinib and ipilimumab did not translate into improved response rates for patients or even a signal of synergistic activity [35].

The tolerability of immunotherapy in sarcomas appears to be similar to other patients. Rash, fever, and fatigue were the most common adverse events. As with other experiences with immunotherapeutic agents, some unusual toxicities were observed necessitating discontinuation of drug and administration of steroids [36]. The Royal Marsden Hospital prognostic scoring system continues to be a valid predictor of survival in a phase 1 trial (Fig. 1) [37]. Our trial had substantially longer overall survival than has been reported in other phase 1 trials of sarcoma patients. Previous trial experiences by our own group as well as groups from the Royal Marsden Hospital and the European phase 1 database have reported consistent OS in the 7.6–9.8 month range with a PFS between 2.1 and 3.5 months [38, 39]. Our extended overall survival with immunotherapy of 13.4 months can be explained either by patient selection for more indolent tumors, since PFS was similar at 2.4 months. Alternatively, there may be some downstream effect of immunotherapy that goes beyond response rates to contribute to an improved overall survival.

It is notable to point out that we experienced one patient with hyper-progression upon initiation of checkpoint blockade (Fig. 3). Others have reported a similar disturbing phenomenon in as many as 9% of patients. This hyper-progression is unrelated to tumor type or burden of disease, but did portend a poor prognosis especially for elderly patients [40]. One group reported that in their experience mutations in MDM2/MDM4 and EGFR predisposed to a hyper-progressor phenotype [41]. Our patient proceeded to another clinical trial where he had continued progression. Ultimately he succumbed to his disease. This reminds us that while adverse events are manageable with immunotherapy there is much we still do not know about the mechanism of these drugs and their ultimate potential.

## Conclusion

In our small and limited retrospective study of various immunotherapies in diverse sarcomas we found encouraging early signals of activity. The strongest clinical responses came from combinations of checkpoint inhibitors. Alveolar soft part sarcomas benefitted particularly well from immune checkpoint inhibitors. Further study and evaluation needs to be done in the heterogeneous and rare group of diseases. Perhaps the biopsy specimens collected from SARC 028 will shed light on mechanisms of response. In addition to understanding the sarcoma immune microenvironment, we need to pursue the early leads in activity found in this study as well as SARC 028 prospective study for patients with high grade undifferentiated pleomorphic sarcoma, dedifferentiated liposarcoma, and alveolar soft part sarcoma.

## Abbreviations

ASPS: Alveolar soft part sarcoma
CR: Complete response
CTCAE: Common Terminology Criteria for Adverse Events
CTLA-4: Cytotoxic T-lymphocyte-associated protein 4
DDLS: Dedifferentiated liposarcoma
GIST: Qastrointestinal stromal tumor
IRB: IInstitutional review board
irRC: Immune-related response criteria
LMS: Leiomyosarcoma
OS: Overall survival
PD1: Programmed cell death protein
PD-L1: Programmed death ligand −1
PFS: Progression-free survival
PR: Partial response
PS: Performance Status
RECIST: Response Evaluation Criteria in Solid Tumors
RMH: Royal Marsden Hospital
SD: Stable disease
TIL: Tumor infiltrating lymphocytes
WDLS: Well-differentiated liposarcoma
